# Childhood Epilepsy with Centrotemporal Spikes: Clinical and Neuropsychological Outcomes 5 Years after Remission

**DOI:** 10.3390/diagnostics10110931

**Published:** 2020-11-10

**Authors:** Costanza Varesio, Martina Paola Zanaboni, Elisa Carlotta Salmin, Chiara Totaro, Martina Totaro, Elena Ballante, Ludovica Pasca, Pierangelo Veggiotti, Valentina De Giorgis

**Affiliations:** 1 Department of Child Neurology and Psychiatry, IRCCS Mondino Foundation, 27100 Pavia, Italy; elisa.salmin@outlook.it (E.C.S.); chiaratot@hotmail.it (C.T.); martina.totaro01@universitadipavia.it (M.T.); ludovica.pasca01@universitadipavia.it (L.P.); valentina.degiorgis@mondino.it (V.D.G.); 2 Department of Brain and Behavioral Sciences, University of Pavia, 27100 Pavia, Italy; 3 BioData Science Center, IRCCS Mondino Foundation, 27100 Pavia, Italy; elena.ballante01@universitadipavia.it; 4 Department of Mathematics, University of Pavia, 27100 Pavia, Italy; 5 Pediatric Neurology Unit, Vittore Buzzi Hospital, 20100 Milano, Italy; pierangelo.veggiotti@unimi.it; 6 Biomedical and Clinical Sciences Department, Luigi Sacco Hospital, University of Milan, 20100 Milano, Italy

**Keywords:** epilepsy with centrotemporal spikes, rolandic, outcome, cognitive, neuropsychological, long-term predictors

## Abstract

Although specific neuropsychological deficits have been recognized during the active phase of epilepsy with centrotemporal spikes (ECTS), the natural cognitive and neuropsychological history after remission has not been elucidated so far. We evaluated the natural cognitive and neuropsychological outcomes five years after disease remission and investigated possible predictors of long-term outcome among socio-demographic and electro-clinical variables. We performed an observational cross-sectional study. Electro-clinical characteristics during the active phase of epilepsy, as well as antiepileptic treatment and premorbid neurodevelopmental concerns were reviewed for 70 patients. At least five years after epilepsy remission, all patients were contacted, and 46 completed a structured questionnaire about patients’ current education and academic skills, general health, and parents’ socio-economic status. Among them, 23 patients underwent an ad hoc cognitive and neuropsychological protocol and emotional-behavioral assessment. Chi-square tests and *t*-tests were carried out to define the role of putative predictors of neuropsychological outcomes. Mean cognitive and neuropsychological performances appeared to be overall adequate, except for the dictation. Positive family history for epilepsy (*p* = 0.01769) and familial socioeconomic status (mother’s schooling (*p* = 0.04169), father’s schooling (*p* = 0.01939), mother’s income (*p* = 0.0262), father’s income (*p* = 0.01331)) were identified as predictors of outcomes. Our data suggest that ECTS with typical electro-clinical features depicts an overall preserved cognitive and neuropsychological long-term outcome. We suggest particular attention should be paid to patients with socio-economic disadvantage and familial history of epilepsy, as they may experience worse neurocognitive post-morbid performances.

## 1. Introduction

Epilepsy with centrotemporal spikes (ECTS), historically referred to as benign epilepsy with centrotemporal spikes (BECTS) or rolandic epilepsy, is the most common focal epilepsy syndrome of childhood, accounting for 15–20% of all childhood epilepsies [1]. It is defined as an age-dependent epilepsy, with a typical onset at 5–8 years [2]. Seizures, which predominantly occur during sleep, are usually stereotyped, self-limiting focal (hemifacial) motor with associated sensory features; awareness is usually retained, but progression to focal to bilateral tonic–clonic seizures is possible, predominantly occurring during sleep [3]. Seizures are overall relatively infrequent, with 60–70% of patients experiencing two to ten seizures lifetime and 10–20% only one. In most cases, it is a self-limiting epileptic syndrome, with seizure remission within adolescence [2]. As suggested by the name, characteristic electroencephalographic (EEG) changes include normal background activity with stereotyped centrotemporal, rolandic spikes followed by slow waves, unilaterally or bilaterally, which can be synchronous or asynchronous on both sides, or migrate from side to side and even extend to adjacent brain regions. The epileptiform activity described may be activated by sleep [4]. Opinions about correct timing and efficacy of treatment are controversial. In a review conducted by Hughes in 2010 [5], it was concluded that two-third of the studies generally favored treatment of ECTS with anti-epileptic drugs (AEDs), and in particular valproic acid (VPA) appeared to be the drug of choice, followed by levetiracetam (LEV).

Despite its overall “benign”/self-limiting course, it is nowadays well established that patients with ECTS may display “atypical” characteristics: early age at onset (<4 years of age), AED side effects, polymorphous seizure types, uncontrolled seizures or history of status epilepticus, daytime seizures, atypical EEG abnormalities or peculiar EEG abnormalities activation during sleep, and the presence of developmental delay or neurologic deficits preceding seizure onset [6,7,8,9]. Moreover, it is nowadays well established that specific transient cognitive and neuropsychological (NPS) deficits may characterize the disease’s active phase [10,11,12]. These findings have led to the hypothesis of a “rolandic epilepsy-related disorders” spectrum, thus comprising conditions with different degrees of severity, from “benign rolandic epilepsy”, as it was once defined, to conditions of “atypical rolandic” epilepsy, up to epileptic encephalopathy conditions such as Landau–Kleffner syndrome and continuous spike and wave during sleep syndrome (CSWS) [10,13]. However, the role of “atypical” features and the presence of NPS deficit at onset has been variably and inconstantly correlated with long-term outcomes both concerning the course of epilepsy and the NPS outcome, making it extremely difficult to identify the role, if any, of potential prognostic predictors of outcomes at the onset. In particular, long term cognitive and NPS outcomes have been objects of debate in recent years, but remain so far to be elucidated [8,14,15].

We in-depth retrospectively electro-clinically characterized patients with a previous diagnosis of ECTS, in remission for five years or more, to obtain a complete cognitive, neuropsychological, and behavioral evaluation after disease remission and identify possible predictors of clinical outcomes.

## 2. Materials and Methods

### 2.1. Patients

This is an observational cross-sectional study involving 70 children with an established diagnosis of ECTS who were regularly followed-up at IRCCS Mondino Foundation between 2004 and 2014. Parents’ written informed consent and patients’ assent were obtained at the time of enrollment in the study. This study was conducted in accordance with the Declaration of Helsinki. The ethics committee of our institute approved the protocol (approval number P-20170023685, approved on 25 September 2017).

Inclusion criteria were defined as follows: (1) established diagnosis of ECTS according to the International League Against Epilepsy (ILAE) definition [16]; (2) patients with or without previous AED treatment were admitted; (3) epilepsy recovery from 5 years or more at the time of the study enrollment. Patients with seizure remission for less than five years, or diagnosed with symptomatic epilepsy, or lost at follow up were excluded from the study.

### 2.2. Data Collection

An ad hoc database (RedCap) was created to collect data. For each patient included in the study, information such as sex, age at seizure onset and offset, seizure semiology, seizure frequency, circadian seizure timing, positive family history for epilepsy, positive personal history for language or neurodevelopment disorder before epilepsy onset, socioeconomic status, comorbidities, type and number of AEDs, and age at therapy introduction/suspension were collected by review of medical records. The reports from all EEGs were reviewed with particular attention to the presence and location of epileptiform activity, slowing, or other background abnormalities. Activation of interictal epileptiform discharges (IEDs) in sleep was categorized as absent, unilateral (left or right), and bilateral.

### 2.3. Follow-Up

Each child was followed with regular outpatient visits during the active phase of the epilepsy until two years after complete seizure recovery and/or suspension of AEDs, if taken.

Five years or more after regular follow-up, all patients were contacted by phone, and 46 (corresponding to 66% of the whole cohort) agreed to complete a structured questionnaire about their current education and academic skills, general health, and parents’ socio-economic status (SES). Three SES levels (high, middle, and low) were calculated based on parents’ professional activities in three classes (manager/high qualification, employee/teacher, or blue-collar) and three educational levels (middle school diploma, high school diploma, and graduation) [17].

### 2.4. Neuropsychological Evaluation

Among the 46 patients contacted by phone, 23 underwent a prospective ad hoc cognitive, emotional, and neuropsychological protocol (fully detailed in Appendix A).

### 2.5. Statistical Analyses

All the analyses were performed with statistical software R, version 3.5.0. Usual descriptive statistics indices (mean, standard deviation), and distributions of absolute frequency and percentage were used to describe the data.

Patients enrolled in the prospective phase who underwent neuropsychological tests were categorized into two groups of outcomes represented by typical (not clinical scores in investigated items) and atypical (at least one score <2 standard deviations in investigated items) neuropsychological profiles.

Comparisons between the groups were obtained by chi-square tests (sex, epilepsy family history, delayed language development, seizure type, seizure frequency, AED administration), and t-tests (age at epilepsy onset, total number of seizures, duration of AED treatment, and total epilepsy).

The statistical significance level was defined with *p* ≤ 0.05, whereas a trend towards significance was defined with *p* between 0.05 and 0.2.

## 3. Results

### 3.1. Clinical Data

Our cohort was composed of 70 patients (44 males and 26 females) with an established diagnosis of ECTS regularly followed-up during the active phase of the epilepsy (mean follow-up 4.6 years, range 3–8 years) (Supplementary Table S1). A positive family history of epilepsy was reported in 14.2% of patients (10 out of 70). A mild prematurity was reported in 6 out of 70 patients (8%). The mean age at epilepsy onset was 7.6 years (range 2–11), with a mean epilepsy duration of 1.6 years (range 0–6). The most common seizure types were focal motor seizures in 58.1% of patients (36 out of 62), followed by focal motor with impaired awareness in 25.8% patients (16 out of 36), and focal to bilateral tonic–clonic in 11.3% (7 out of 36). It was not possible to reconstruct the seizures’ semiotics of the remaining 11 patients. Seizures were overall relatively infrequent. Patients experienced a mean of 5.72 seizures lifetime (range 1–20) with an annual frequency in 25% (15 out of 59), six-monthly frequency in 37% (22 out of 59), monthly in 23% (14 out of 59), and daily in 5% of patients (3 out of 59). It was not possible to reconstruct the seizures’ frequency in the remaining 11 patients.

The background EEG activity while awake was normal in 92.8% of patients (65 out of 70), and moderately abnormal in 7.2% (5 out of 70) of patients. Interictal EEG during sleep showed the presence of IEDs in all patients with bilateral rolandic discharges in 20% of patients (13 out of 64), unifocal rolandic right discharges in 43% (28 out of 64 patients), and unifocal rolandic left discharges in 36% (23 out of 64 patients) of patients. It was not possible to obtain a sleep EEG in the remaining six patients.

As far as anti-epileptic treatment is concerned, information about therapy in 11 patients was lacking; 49% of patients (29 out of 59) never took AEDs. The remaining patients were administered monotherapy with valproic acid (13 out of 30 patients), clobazam (6 out of 30), levetiracetam (1 out of 30), carbamazepine (2 out of 30), and oxcarbazepine (4 out of 30). Four patients were treated with poly-therapy. The average duration of therapy was 4.23 years (range 1–11 years).

### 3.2. Health, Academic Performances, and Familial SES after Remission

At least five years after epilepsy remission, 46 patients’ proxies underwent phone interviews to detail their general health, academic performances, and familial SES.

Parents reported patients’ high academic performances in 60% of cases (26 out of 43), middle school performances in 23% of cases (10 out of 43), and low academic performances in 16% of patients (7 out of 43). Three patients did not answer about academic performances. Overall good general health with no chronic diseases was reported at the current time for all patients. SES was reported as high in 19% of patients (9 out of 46), middle in 56% of patients (26 out of 46), and low in 24% of patients (11 out of 46).

### 3.3. Neuropsychological and Behavioral Assessment after Remission

#### 3.3.1. Neuropsychological Profile

Twenty-three patients underwent an ad hoc perspective cognitive and neuropsychological protocol and emotional, behavioral assessment (Table 1 and Table 2).

The mean Intelligent Quotient (IQ) measured by Wechsler Intelligence Scale was 97.74 ± 20.45, indicating a normal cognitive performance. In detail, the mean Verbal Comprehension Index (VCI) was 100.91 ± 16.34, the mean Perceptual Reasoning Index (PRI) was 102.78 ± 18.06, the mean Working Memory Index (WMI) was 92.22 ± 19.31, and the mean Processing Speed Index (PSI) was 94.17 ± 19.12.

The following neuropsychological domain-specific tests were taken into consideration:

1. In visuomotor and visuospatial skills (VMI developmental test), mean normal scores were obtained in all subtests (visuomotor integration, visual perception, motor coordination).
2. In attention and executive functioning (Nepsy II) domains, mean results within the normal range were obtained in visual and auditory attention and inhibition. Borderline mean results were recorded in response tests, graphic fluency, rapid naming, and cognitive flexibility.
3. In language skills (Nepsy II and BVN 12–18) mean normal scores were obtained in lexical denomination, instruction understanding, and semantic fluency, whereas mean borderline results were reached in the phonological fluency test.
4. In memory and learning skills (Nepsy II), mean normal scores were obtained in all subtests (immediate list memory, deferred list memory, immediate design memory, deferred design memory).
5. In academic skills (DDE-2, MT, BDE-2, BVSCO-2 tests), mean normal scores were recorded in all tests (reading, writing, comprehension, calculation, graphomotor fluence), except for dictation, where a mean pathological result was obtained.

#### 3.3.2. Behavioral Assessment (Table 1 and Table 2)

From the behavioral perspective, our population demonstrated the absence of risk indicators assessed through a standardized diagnostic tool (Child Behavior Check List). Test mean results were below the pathological range (pathological T scores >70); internalizing problems (anxiety, depression, withdrawn-depression, and somatic complaints) showed mean T-score 55.85 ± 9.03, externalizing problems (rule-breaking and aggressive behavior) showed mean T-score 50.93 ± 12.22, and the total score was 53.24 ± 11.55.

#### 3.3.3. Predictors of Neuropsychological Outcome

**Figure 1:**
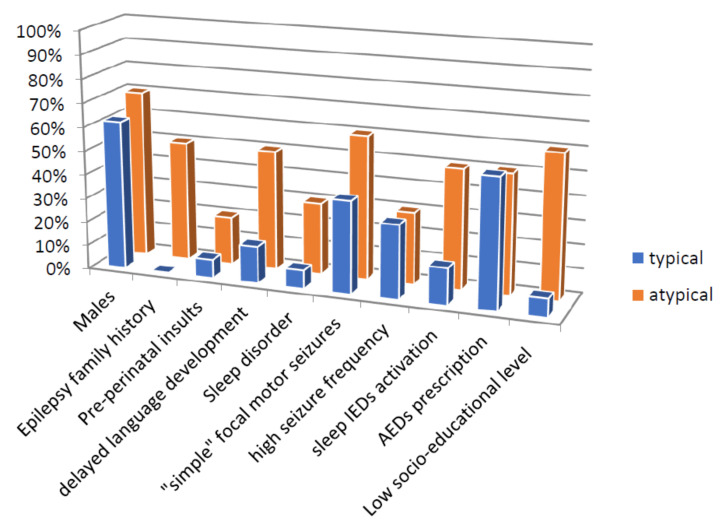
Socio-demographical and electro-clinical differences in the two groups of neuropsychological outcome.

Differences in electro-clinical and socio-demographic features between the two groups of typical and atypical neuropsychological outcomes were tested to define the role of putative predictors of neuropsychological outcomes in our sample (Table 3, Figure 1). Thirteen patients fulfilled the “typical” neuropsychological profile group, with adequate overall scores in investigated items. Ten patients were classified as the “atypical” neuropsychological profile group because they obtained a principal diagnosis of intellectual disability (*n* = 3), specific learning disability (*n* = 3), language disorder (*n* = 2), autistic disorder spectrum (*n* = 1), and attention deficit (*n* = 1) combined with other neuropsychological impairments such as attention and memory deficit and aspecific learning disorder. Among them, 50% of patients (5 out of 10) had a positive personal history of language disorder in the first years of life.

Significant differences were found for the following characteristics:

- Positive family history for epilepsy: 5 out of 10 patients in the “atypical” group versus 0 out of 13 patients in the “typical” group (*p* = 0.01769).
- Familial SES: A better SES level was reported in the “typical” (1 high, 11 middle, 1 low level) compared to the “atypical” group (0 high, 3 middle, 7 low level). In particular, higher mother’s schooling (*p* = 0.04169), higher father’s schooling (*p* = 0.01939), higher mother’s income (*p* = 0.0262), and higher father’s income (*p* = 0.01331) were obtained in the “typical” compared to the “atypical” group.

It is also worth mentioning the following results that did not obtain a statistical significance but a trend towards significance (*p* < 0.2):

- IED activation in sleep: higher IED activation in sleep EEG recordings was found in the “atypical” as compared to the “typical” group. In particular, 5 out of 10 patients in the “atypical” group had a generalized IED activation in sleep; conversely, 2 out of 13 patients in the typical group had the same EEG pattern (*p* = 0.09748).
- Mean AED treatment duration: in the “typical” group, a lower AED treatment duration was observed (mean 3.5 years) as compared to the “atypical” group (mean of 5.6 years) (*p* = 0.1027).
- Presence of language disorder before epilepsy onset: in the “typical” group, 2 out of 13 patients presented delayed language development as compared to 5 out of 10 in the “atypical” group (*p* = 0.183).

Detailed results of the statistical correlations are reported in Table 3.

## 4. Discussion

The present paper reports the findings of a cross-sectional observational study on patients with a previous diagnosis of ECTS, recovered for at least five years, aimed at both retrospectively evaluating electroclinical data and assessing general outcomes after epilepsy remission. In a subset of this population, the neuropsychological profile was explored after epilepsy remission, searching for socio-demographic and clinical variables related to the long-term outcomes.

The electro-clinical data of our cohort of 70 patients appeared overall in line with previous reported epidemiological data concerning the mean age at epilepsy onset of 7 years [12,18], the epileptological characteristics (prevalence of focal motor seizures without consciousness impairment), low lifetime seizure frequency (half-yearly in 37% of patients), and disease duration of about two years (range 0–6) [2,8]. About half of our patients (51%) were administered at least one AED, with an average therapy duration of about four years. This datum differed from what was reported in other long-term outcome studies, where the percentages of patients treated with AEDs were higher, around 80% [8,11,15]. This difference can be read in light of two considerations: the first concerns the enrollment in the study of patients at the most “benign” end of the ETCS spectrum; the second, as a direct consequence, could lie in the fact that, according to the clinical practice in use at our center, AED treatment is usually reserved to patients with high seizure frequency, focal seizures with impaired consciousness or focal to bilateral seizures, or daytime seizures. The AED of choice was mainly valproic acid, in line with the standard therapeutic choice at the European level [19].

EEG features in our sample appear to be consistent with the typical observation of high voltage spikes and spikes and waves in the centrotemporal region in the context of an overall normal background activity and organization. An increase in the rate of epileptic discharges during drowsiness and NREM sleep is a common feature of the syndrome [20,21,22]. In our sample NREM sleep activation of epileptic discharges was observed in almost all patients, unifocal (right or left) in 79% of patients, and bilateral in 21% of patients.

The “benignity” of ECTS in cognitive and neuropsychological profiles has been an object of debate. Although many studies have emphasized the presence of specific and transitory deficits in almost all neuropsychological domains during the active phase of epilepsy [23,24,25,26,27,28,29], the duration of these deficits has not been clearly defined so far.

In our sample of 46 surveyed patients, proxy-reports suggested that five years after remission, the global cognitive and academic skills were overall preserved; in almost 83% of our sample, school performances were reported as good or discrete compared to peers. Moreover, in the subset of 23 patients tested five years after ECTS remission, average cognitive performance was within the normal range.

Similarly, patients obtained average normal scores in nearly all neuropsychological specific domains. Our results align with what is observed in other studies focusing on long term ECTS natural history [8,14,15], thus confirming an overall good prognosis.

However, a deficit in piece dictation was observed in the field of academic skills (7/23), which were, for the rest, preserved. This competence could be read in light of their impairment in language performance and executive functions, which may have affected the test results. We indeed observed fragility in the field of language, and in particular in the phonological fluency test, as already observed in previous studies conducted after ECTS remission [30].

Borderline average scores were also observed in visuospatial attention and executive function skills (namely response tests, graphic fluency, rapid naming, and cognitive flexibility), as observed in previous studies [29,31,32].

Even with the limitations represented by low sample size and by the absence of an NPS assessment at epilepsy onset, we can speculate that these deficits and fragility may be the result of more frank disturbances typical of the first phase of the disease, transient but crucial for long-term neuropsychological development, as previously described in the literature [29,33,34,35,36].

In the literature, the atypical ECTS trajectory is interpreted in light of the electroclinical and neuropsychological characteristics at onset (i.e., early age, seizure types, IEDs sleep activation, neurocognitive deficit). In our study, we defined a blown neurocognitive deficit in the long-term follow-up as a criterion for atypicality. Thus, we divided our cohort based on neuropsychological outcome. Typical patients obtained overall adequate scores at the neuropsychological evaluation; atypical cases had at follow-up a diagnosis of intellectual disability, specific learning disorders, language deficit, autism spectrum disorder, and attention deficit.

Neuropsychological findings in the two groups were then correlated to socio-demographical and electro-clinical variables to define the role of putative long-term predictors. In line with Callenbach and colleagues [8], our data support previous observations that none of the examined disease course electro-clinical variables seemed to be a strong predictor of long-term disease course and neuropsychological outcomes except for a familiar positive history for epilepsy, which appeared to be strongly related to an atypical neuropsychological outcome.

Since we realized that our cohort is relatively small, we were well aware that one or more putative predictors of outcomes could have reached statistical significance in the presence of a larger sample.

Therefore, we believed it important to underline that a trend towards significance was found for frequent epileptiform discharges in NREM sleep (without configuring a picture of CSWS) and worse evolution. The presence of frequent centrotemporal spikes during sleep has been regarded as an important potential predictor of adverse neuropsychological outcomes [11] and, in particular, in the fields of language [37], learning disorders [26], and attention skills [28]. Vannest and colleagues [2] suggested that during the active phase of epilepsy, the brain develops structurally and functionally, and epileptic seizures and epileptic discharges may alter this development. In line with these observations, a trend towards significance was found for language impairment before epilepsy onset, thus supporting the hypothesis of a common pathogenic pathway. It should be noted that the presence of language impairment preceding epilepsy onset was observed in almost one-third of our sample, thus supporting the previous hypothesis by Overvliet and colleagues [38] that language impairment could be regarded as a precursor rather than a consequence of ETCS.

The role of AEDs in cognitive outcomes still needs to be clarified [2]. Based on the data collected in this study, it cannot be excluded that a longer duration of therapy (*p* < 0.2) could have negatively affected the neuropsychological and cognitive results; larger case series are needed to elucidate this aspect.

Predictors of outcomes have been found among socio-demographic variables; the socio-economic disadvantage seems to play a role in setting the risk of atypical long-term neuropsychological outcomes. This observation is in line with what was noticed in previous studies by Callenbach and colleagues [8] and Massa and colleagues [39], who described an association of social–familial complications with long-lasting neuropsychological impairments.

A possible explanation for this finding could be linked to parents’ lower economic and social possibilities with lower education and income, offering a limited cognitive stimulation in general. Then, in those families, in the presence of a predisposing condition such as epilepsy, access to neuropsychological rehabilitation could hardly be achievable. Since cognitive and neuropsychological functions are still developing in children, the long-term outcomes also depend on early rehabilitation and psycho-educational intervention, as observed by Filippini and colleagues [29].

Although ECTS is amongst the most commonly encountered and relatively benign epilepsy syndromes affecting children, it still poses many “treats” for clinicians. The observation that the long-term outcomes appear to be worse in patients from fragile families underlines the importance of family-centered care in children with neuropsychiatric disorders and in particular with epilepsy [40]. Such an approach can bring potential and synergic benefits related to the various patient- and family-centered care dimensions (not only limited to epilepsy itself), which can maximize patients’ management and long-term outcomes.

This study has limitations that should be considered in the interpretation of findings. First, the sample of patients prospectively enrolled is considerably lower than the potentially enrollable population; only 65% of families agreed to undergo the telephone interview, and only a third of patients underwent a complete cognitive and neuropsychological evaluation. This low respondent rate appeared to be in line with observations of a progressive decline of willingness to participate in epidemiological studies, especially when the youth population is concerned, thus making it a challenge to recruit a sample that is representative of the entire population [41]. In this regard, we believe it is crucial to extend the study, enrolling a more significant number of patients, through multicenter studies.

We hypothesized this denial to take part in the prospective study could have at least two explanations: the first relies on the fear of retracing the perceived stigma of being socially discriminated because of the disease, even if in remission; and the second is related to the perception of not gaining any advantage from participating in the study. Moreover, our prospective clinical information is mainly based on proxy-reports, based on phone calls, which may have limited value, not based on direct observation. Patients who accepted to undergo full assessment after disease remission do not have neuropsychological data at epilepsy onset and during follow up, thus making it impossible to trace trajectories of cognitive and neuropsychological development.

## 5. Conclusions

Overall, our data suggest that ECTS with typical disease course electro-clinical features depicts overall preserved cognitive and neuropsychological long-term outcomes after remission. With this work, we have shed further light on long-term neurocognitive predictors of outcomes. Although no decisive predictive factors could be identified among epilepsy variables, we suggest particular attention should be paid to patients with socio-economic disadvantage and familial history of epilepsy as they may determine worse neurocognitive post-morbid performances. We can suggest that patients with a positive family history of epilepsy should undergo genetic counseling to evaluate the opportunity to investigate genetic susceptibility to epilepsy.

Finally, we believe that it is essential to carry out cognitive and neuropsychological assessments at the time of diagnosis and during the follow-up to trace the trajectories of neuropsychological development of these patients more deeply.

## Figures and Tables

**Table 1:** Cognitive and neuropsychological test results in the 23 perspective evaluated patients.

Domain	Test/Subtest-Index	Mean (SD)	Level
Intellectual functioning	Wechsler Intelligence Scale for children (full-scale IQ) ^a^	97.74 (20.45)	Normal
	Verbal Comprehension Index (VCI) ^a^	100.91 (16.34)	Normal
	Perceptual Reasoning Index (PRI) ^a^	102.78 (18.06)	Normal
	Working Memory Index (WMI) ^a^	92.22 (19.31)	Normal
	Processing Speed Index (PSI) ^a^	94.17 (19.12)	Normal
Language	Picture naming ^b^	−0,47 (1.34)	Normal
	Comprehension of instructions ^a^	8,04 (3.71)	Normal
	Word generation		
	Semantic ^a^	9.13 (3.91)	Normal
	Phonemic ^a^	7.35 (2.77)	Mildly impaired
Attention and executive functioning	Visual attention ^a^	9.61 (4.47)	Normal
	Auditory attention ^b^	−0.61 (2.32)	Normal
	Response set ^b^	−2.2 (7.97)	Severely impaired
	Design fluency ^a^	7.83 (2.81)	Mildly impaired
	Inhibition naming ^a^	7.5 (2.92)	Mildly impaired
	Inhibition ^a^	8 (3.09)	Normal
	Switching ^a^	7.18 (2.52)	Mildly impaired
Visuomotor and visuospatial skills	VMI ^b^	88.48 (12.63)	Normal
	Visual perception ^b^	94.87 (14.38)	Normal
	Motor coordination ^b^	86.09 (14.73)	Normal
Memory and learning	List memory		
	Immediate memory ^a^	8.83 (2.59)	Normal
	Delayed memory ^a^	8.35 (3.59)	Normal
	Total memory ^a^	8.52 (2.63)	Normal
	Memory for designs		
	Immediate memory ^a^	6.48 (4.57)	Mildly impaired
	Delayed memory ^a^	8 (4.36)	Normal
Academic skills	Word reading		
	Rapidity sill/sec ^b^	−0.53 (1.36)	Normal
	Accuracy ^b^	0.57 (1.82)	Normal
	Non-word reading		
	Rapidity ^b^	−0.62 (1.11)	Normal
	Accuracy ^b^	0.2 (1.36)	Normal
	Reading test		
	Rapidity sill/sec ^b^	−0.87 (1.66)	Normal
	Accuracy ^b^	0.56 (1.86)	Normal
	Reading comprehension test ^b^	−0.46 (1.1)	Normal
	Dictations of words and pseudowords ^b^	0.74 (3.68)	Normal
	Dictations of pseudowords ^b^	−0.28 (1.76)	Normal
	Text dictation ^b^	3.88 (9.55)	Severely impaired
	Handwriting speed		
	Task 1 ^b^	−0.57 (1.42)	Normal
	Task 2 ^b^	−0.99 (1.49)	Normal
	Task 3 ^b^	−1.11 (1.47)	Mildly impaired
	Mathematics competence		
	Number Index ^b^	103.41 (22.89)	Normal
	Calculation Index ^b^	91.27 (27.81)	Normal
	Sense of Number Index ^b^	100.64 (23.4)	Normal
	Total Index ^b^	97.45 (24.48)	Normal
Behavioral and emotional functioning	Internalizing problems ^c^	55.85 (9.03)	Normal
	Externalizing problems ^c^	50.93 (12.22)	Normal
	Total problems ^c^	53.24 (11.55)	Normal

M: mean, sd: standard deviation; ^a^ age-adjusted scaled scores, mean = 10, SD = 3; ^b^ age-adjusted standard scores, mean = 100, SD = 15; ^c^ age-adjusted T-scores, mean = 50, SD = 10.

**Table 2:** Results interpretation according to standardized scores.

	z-Scores	Scaled Score	T Score	Standard Score
Normal or typical	>−0.99	>8	<65	85–114 normal
Mildly impaired	−2 and −1	7–6	65–70	70–84 borderline
severely impaired	<−2 as	<5	>70	<70 deficit

**Table 3:** Socio-demographical and electro-clinical differences in the two groups of neuropsychological outcome. Typical patients were those who obtained fully normal scores at the neuropsychological evaluation. Atypical cases obtained diagnosis of intellectual disability, autism spectrum disorder, specific and aspecific learning disorders. The socio-demographic and clinical variables were considered statistically significant predictors of outcome if *p*-value ≤ 0.05 **, a trend toward significance was still considered (*p*-value ≤ 0.2 *).

	Typical *n* = 13	Atypical *n* = 10	*p*-Value
Males	8/13	7/10	1
Pre-perinatal insults	1/13	2/10	0.4919
Epilepsy family history	0/13	5/10	0.01769 **
Delayed language development	2/13	5/10	0.183 *
Sleep disorders	1/13	3/10	0.5812
Age at epilepsy onset (mean)	6	5.9	0.8019
Epilepsy duration (mean)	2	1.9	0.8015
Total seizures during follow-up (mean)	4	6	0.7606
“Simple” motor focal seizures	5/13	6/10	0.5683
High seizure frequency	4/13	3/10	0.4773
Sleep IEDs activation	2/13	5/10	0.09748 *
AEDs prescription	7/13	5/10	1
AEDs duration (years)	3.5	5.6	0.1027 *
Low educational level			
Father	1/13	6/10	0.01939 **
Mother	1/13	5/10	0.04169 **
Low income bracket			
Father	0/13	5/10	0.01331 **
Mother	2/13	7/10	0.02626 **

Abbreviations: *n* = number; AED = antiepileptic drug; high seizure frequency = ≥ monthly.

## Supplementary Materials

The following are available online at https://www.mdpi.com/2075-4418/10/11/931/s1, Table S1: Summary of all the clinical characteristics of the study population.

## Appendix A: Neuropsychological Assessment

### Appendix A.1. Intellectual Functioning

The cognitive baseline was measured with WISC IV [42] or WAIS IV [43] scale that give five main scores for index abilities and Full Scale IQ. Full Scale IQ correspond from four factors: Verbal Comprehension Index which measures verbal abilities, verbal reasoning; and to use language to solve novel problems. Perceptual Reasoning Index: a measure of an individual’s ability to understand visual information and abstract problem solving. Working Memory Index: A measure of an individual’s ability to hold verbal information in short-term memory and to manipulate verbal material for a short period of time Processing Speed Index: A measure of mental speed and focus attention on a task. Full Scale IQ: A measure of an individual’s overall cognitive ability based on the individual’s performance on all the subtests.

### Appendix A.2. Domain Language

In the domain of language, Picture Naming test [44], Comprehension of Instructions [45] and Word generation [4] were used for the assessment.

Picture Naming Test is designed to assess lexical abilities, it consisting of 88 black and white line drawings of objects and the child is asked to denominate the figures.

Comprehension of Instructions is designed to assess the ability to perceive, process, and execute oral instructions of increasing syntactic complexity. The child points the correct picture in response to examiner commands.

Word generation is used to assess verbal productivity through the ability to generate words within specific semantic and initial letter categories in 60 s.

### Appendix A.3. Domain Attention and Executive Functioning

In the domain of attention and executive functioning: Visual attention [45], Auditory Attention and Response [45], Design Fluency [45] and Inhibition [45] were used for the assessment

Visual Attention is designed to assess visual selective and divided attention on two more or less difficult parts.

Auditory Attention is designed to assess selective auditory attention and vigilance. Response Set, the second part of the subtest, it’s designed to assess the ability to shift and maintain a new and complex set involving both inhibition of previously learned responses and correctly responding to matching or contrasting stimuli. The child listens to a series of words and touches the appropriate circle when he or she hears a target word.

Design Fluency is a nonverbal fluency task, the test is designed to assess the child’s ability to generate unique designs by connecting up to five dots, in a given time limit from both structured and unstructured dot arrays.

Inhibition is designed to assess rapid naming, the ability to inhibit automatic responses in favor of novel responses and the ability to switch between response types. The child looks at a series of black and white shapes or arrows and names either the shape or direction or an alternate response, depending on the color of the shape or arrow.

### Appendix A.4. Domain Visuomotor and Visuospatial Skills

The visuomotor integration [VMI] developmental test [46] were used to assess fine motor speed and precision. The test consists of drawings of geometric forms arranged in order of increasing difficulty that the child is asked to copy.

### Appendix A.5. Domain Memory and Learning

Memory and Learning domain are List Memory, Memory for Designs [45].

The subcomponents assessed include working memory and immediate and delayed memory for abstract designs and lists.

List memory and List Memory Delayed is designed to assess verbal learning and memory.

The child is read a list of words several times, then an interference list and the child is asked to recall them after each presentation. A delayed task assesses long-term memory for words.

Memory for Designs and Memory for Designs Delayed is designed to assess visuospatial memory for novel visual material. The child is shown a grid with four to ten designs on a page, which is then removed from view. The child selects the designs from a set of cards and places the cards on a grid in the same location as previously shown. A delayed task assesses long-term visuospatial memory.

### Appendix A.6. Domain Academic Skills

Academic skills reading [47,48,49,50,51], writing [47,52,53], comprehension [49,50,51,52] and arithmetic [54] ability were assessed.

Reading levels has been investigated through lists of words, nonwords and story. The subject is asked to read aloud and as fast as possible four lists of words and three lists of nonwords nad a story. Performance is evaluated in terms of both accuracy (number of errors) and speed (number of syllables per second).

In the reading comprehension task the child is asked to read a story and then to choose the correct response between some alternatives.

In the writing test, one list of words, one list of nonwords and a story are dictated. Accuracy [number of errors].

The praxis of writing was examined using three writing tasks: (a) writing the sequence of letters “le” for 1 min; (b) writing the sequence of letters “uno” for 1 min; and (c) writing the sequence of numbers “uno-due-” and so on for 1 min. The test involved calculation of the measure of fluency, as how many graphemes were written correctly in 1 min.

The mathematical performance was examinated through a battery which is divided into three areas numerical processing, with counting and numerical transcoding tasks; calculation, with tests which assess the ability to process algebra symbols, retrieve arithmetic facts and perform written and mental calculations;sense of number, with tests which investigate semantic aspects of numerical processing and calculation.

### Appendix A.7. Domain Behavioral and Emotional Functioning

The Child Behavior Checklist (CBCL) [55] is a widely used questionnaire to assess behavioral and emotional problems. The questionnaire aimed to identify the presence of internalizing problems (anxiety, depression, withdrawn-depression and somatic complaints) and externalizing problems (rule-breaking and aggressive behavior).
